# Diagnosis and Treatment Challenges of an Atypical Case of Pemphigus vulgaris During the COVID-19 Pandemic

**DOI:** 10.7759/cureus.21739

**Published:** 2022-01-30

**Authors:** Mădălina Laura Banciu, Codruta Dobrica, Ana Maria Malciu, Cristina Vâjâitu, Vlad Mihai Voiculescu

**Affiliations:** 1 Dermatology, Elias Emergency University Hospital, Bucharest, ROU; 2 Pathophysiology, University of Medicine and Pharmacy of Craiova, Craiova, ROU; 3 Dermatology, Carol Davila University of Medicine and Pharmacy, Bucharest, ROU

**Keywords:** pemphigus foliaceus, pemphigus vulgaris, sars-cov-2, dapsone, rituximab, targeted therapy

## Abstract

Pemphigus defines a group of rare autoimmune blistering diseases that affect the skin and mucous membranes, with pemphigus vulgaris being the most common form that has increased morbidity and mortality in the absence of an early diagnosis and treatment.

We report the case of a 24-year-old male with an atypical form of pemphigus vulgaris with cutaneous onset and subsequent involvement of the oral cavity. The management of the patient initially consisted of long-term systemic corticosteroid therapy. Following a mild form of SARS-CoV-2 infection and a flare-up of the disease in this context, which was not controlled with high doses of systemic corticosteroids, targeted therapy with rituximab was initiated but immediately stopped due to the manifestations of urticaria and angioedema. Considering the magnitude of these reactions, dapsone systemic therapy i.e., a steroid-sparing agent with minimal risk of infections, was started and managed to control the underlying disease.

The management of this case of pemphigus vulgaris was challenging for both the patient and his physician, as the patient developed COVID-19 which caused disease complications and implied additional costs. This case highlights the importance of an accurate diagnosis given the atypical onset of the disease and the financial limitations with the impossibility of performing all confirmatory diagnostic tests.

## Introduction

Pemphigus (derived from the Greek term ‘pemphix’, which means blister) defines a group of rare chronic autoimmune diseases that affect the skin and mucous membranes with severe impairment of the quality of life. From a pathogenic point of view, the disease is characterized by the presence of immunoglobulin G (IgG) antibodies directed against intercellular adhesion molecules in the structure of desmosomes, i.e., desmoglein 1 and/or 3, major components of desmosomes causing acantholysis and the development of blisters and erosions [1]. The epidemiology of the disease varies with the geographical region and has an average age of diagnosis between 50 to 60 years [2].

The most prevalent types of pemphigus observed clinically are pemphigus vulgaris and pemphigus foliaceus. Several uncommon forms have also been reported in recent years, such as paraneoplastic pemphigus, herpetiform pemphigus, and immunoglobulin A (IgA) pemphigus. Pemphigus vulgaris is the most common form of pemphigus, accounting for nearly 70% of cases [3]. It has a mortality rate between 60% to 90% in the absence of an accurate diagnosis established in a timely manner, due to hydro-electrolytic imbalances, septic potential, and multiple organ failure including renal and cardiac failure [4]. In clinical practice, 50% to 70% of the patients with pemphigus vulgaris initially present with mucosal lesions, particularly oral lesions, with subsequent involvement of the skin [5].

The disease’s fatal potential requires a rapidly established treatment that consists of two stages that aim to decrease the formation of autoantibodies: the phase of clinical control of the disease which includes the healing of the existing lesions and the prevention of the development of new blisters within two weeks and the consolidation phase of therapy [6].

## Case presentation

A 24-year-old man, an active smoker without a family history of skin diseases or a notable medical history, and without any previous or ongoing medication, presented to the dermatology clinic for blisters and post-bullous erosions on the skin and oral cavity which had been evolving for one year.

**Figure 1:**
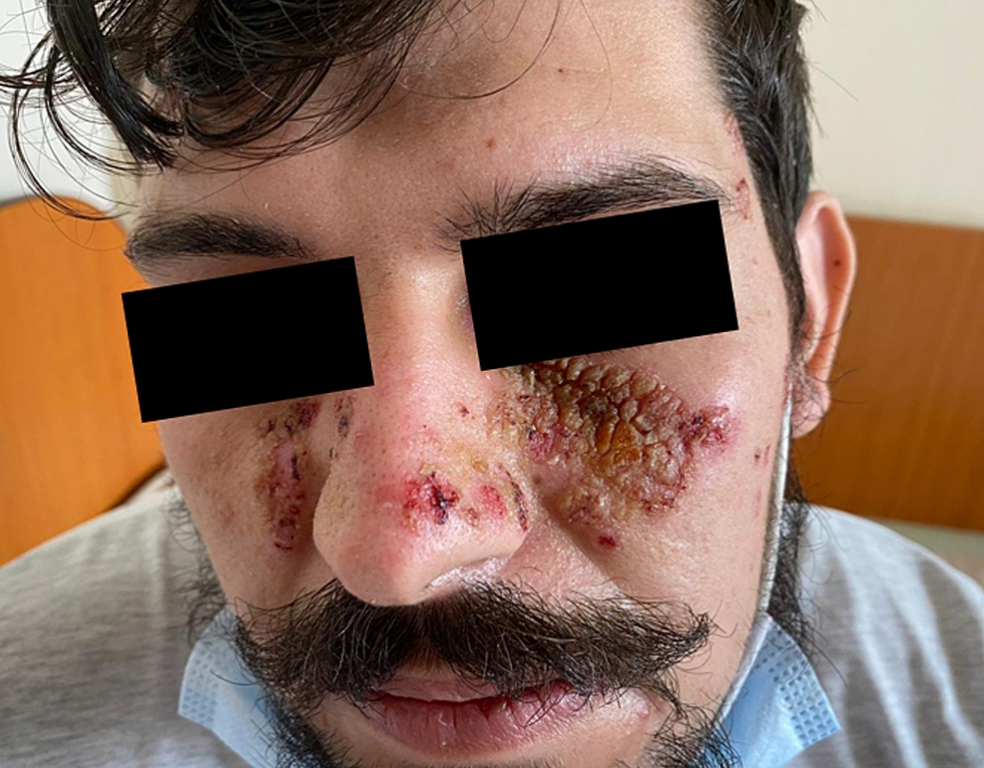
Erosions with malar distribution

**Figure 2:**
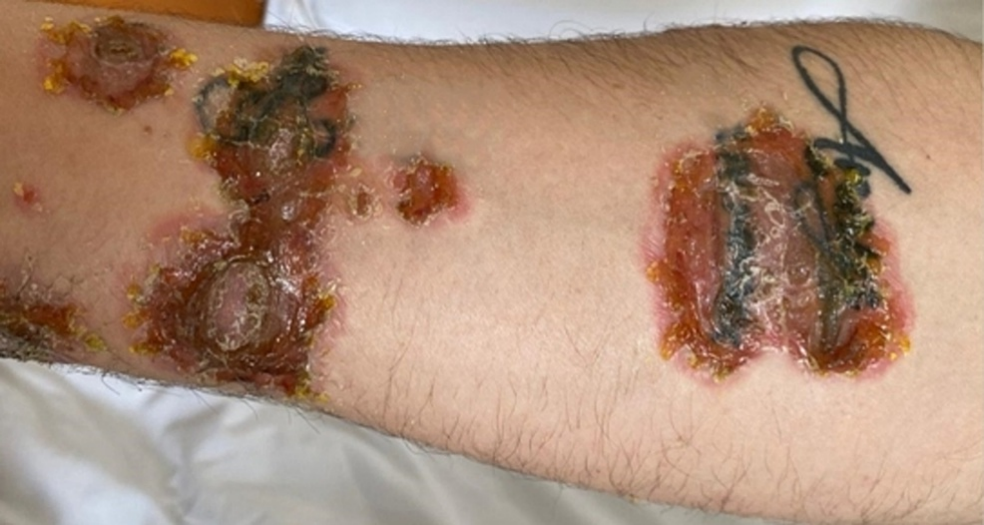
Erosions covered by sero-hematous crusts

**Figure 3:**
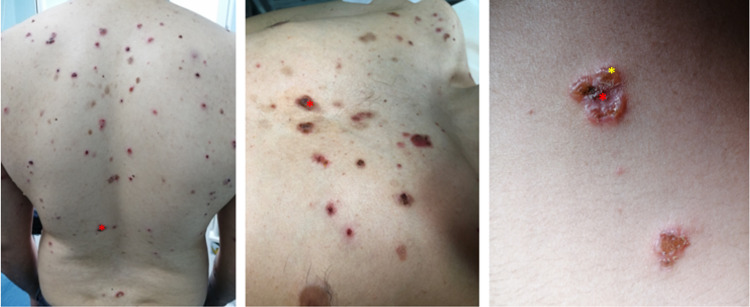
Post-bullous erosions (red asterisk) centered by sero-hematous crusts with blisters in the periphery (yellow asterisk) on the seborrheic skin areas

The clinical examination revealed erosions covered by sero-hematous crusts with malar distribution, similar to the "malar rash" (Figure 1), and also post-bullous erosions centered by sero-hematous crusts with blisters in the periphery on the seborrheic skin areas (Figure 2, Figure 3), evolving for one year. Firm sliding pressure of the skin resulted in exfoliation of the outermost layer, resulting in erosions and indicating a positive Nikolsky sign. The patient also displayed painful oral mucosal erosions with subsequent onset a few months after the development of the cutaneous ones. The first lesions developed in March 2020 and the first presentation to the dermatologist was in January 2021 when he received systemic and topical antibiotic therapy. As the lesions did not heal during this treatment, the patient returned to the dermatology clinic in February 2021.

**Figure 4:**
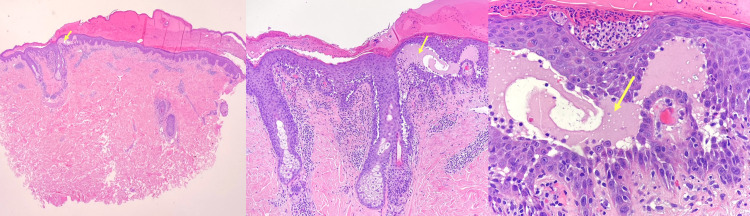
Photomicrograph (x20, x100, x400, hematoxylin and eosin stained) showing suprabasal acantholysis (yellow arrows)

The laboratory work-up only detected a slight leukocytosis due to the superinfection of the lesions. The histopathological examination of the skin biopsy revealed suprabasal acantholysis that extended to the cutaneous annexes, typical changes of pemphigus vulgaris (Figure 4).

Patient management and follow-up

Considering the clinical and laboratory data, systemic antibiotic therapy and corticosteroids were initiated during the hospital stay, followed by long-term, slowly tapered steroid therapy after the patient’s discharge. Three months later, at the reassessment, except for some residual lesions, no new lesions were noted. At about one week after the reassessment presentation, the patient was diagnosed with a mild form of SARS-CoV-2 infection during which he experienced an exacerbation of the disease with the development of new bullous lesions. At the time of COVID-19 diagnosis, the patient was still on systemic corticosteroids for his underlying disease (the dose was slowly tapered reaching half of the initial dose decided at the first hospitalization). Given the worsening of the disease in the context of COVID-19, we decided to escalate the dose of systemic corticosteroids. At two months post-SARS-CoV-2 infection, in the absence of therapeutic response to high doses of corticosteroids, we considered initiating therapy with rituximab. Thus, the patient received premedication (oral desloratadine and acetaminophen, and intravenous methylprednisolone) and systemic therapy was initiated. However, the patient developed in a short time a severe urticarial skin reaction and typical manifestations of angioedema. Considering the severe allergic response, after the allergy assessment and the clinical stabilization of the patient, we decided to stop the therapy with rituximab and to continue systemic corticosteroids in combination with dapsone. The choice of dapsone as a cortico-sparing agent was mainly influenced by the long-term systemic effects of immunosuppressive therapy and by the inability to administer iv immunoglobulins due to financial reasons. The patient had received the combination of dapsone and low doses of systemic corticosteroids for five months, with the subsequent complete elimination of corticosteroids from the treatment regimen, with no signs of disease reactivation.

## Discussion

This case has many peculiarities related to clinical presentation and management options.

The onset of lesions occurred at the age of 23 in contrast to data from the literature according to which, the average age of onset of pemphigus vulgaris lesions is of about 50 to 60 years and with a female predominance [7].

The atypical onset of the lesions i.e., cutaneous with subsequent involvement of the mucous membranes, raised the suspicion of pemphigus foliaceus and initially masked the underlying pathology. A transition or an association between the two forms can be discussed as it has been rarely described in the literature. But we cannot prove this by the lack of immunofluorescence. As the patient belonged to a disadvantaged social category with limited financial possibilities, the patient could only undergo the histopathological examination of the lesion at the time of the first presentation in the clinic which revealed suprabasal acantholysis and determined the initiation of life-saving systemic therapy. Much more sensitive than conventional histopathology would have been direct immunofluorescence, showing intercellular immunoglobin G (IgG) deposits and C3 complement in the epidermis. As alternatives, indirect immunofluorescence (IIF) microscopy of serum antibody titers or specific enzyme-linked immunosorbent assays (ELISA) could have been used. These are not gold standards, but can indicate more rapidly the possibility of a diagnosis of pemphigus until histopathological confirmation and direct immunofluorescence result [3]. In the skin and mucous membranes, there is a different expression of desmogleins 1 and 3. In the skin, desmoglein 1 is more expressed in the superficial layers, while desmoglein 3 is in the deeper layers of the epidermis. In the mucous membranes, desmoglein 3 is more expressed than desmoglein 1. Given this, antidesmoglein 1 antibodies will cause superficial blisters in the skin, but not in the mucous membranes because at that level desmoglein 3 compensates for the loss of desmoglein 1 (compensation theory) [8]. The transition between the two forms of pemphigus is a rare phenomenon. However, data from the literature showing that the transformation of vulgar pemphigus into foliaceus pemphigus is more frequent than vice versa [9]. The mechanism of transition between pemphigus could be the phenomenon of "epitope spreading", an autoimmune or inflammatory response that causes tissue damage with exposure to initially hidden proteins, and secondary autoimmune response. In the etiopathogenesis of pemphigus foliaceus, the central element is the presence of anti-desmoglein 1 antibodies. But in the case of transition of pemphigus foliaceus to pemphigus vulgaris, in the context of exacerbation of the disease, initially hidden antigens such as desmoglein 3 may be exposed, causing a change in the disease phenotype. This phenomenon, rarely described in the literature, based on the qualitative changes in desmoglein antibody profile, has clinical and therapeutic implications. Despite being clinically and anamnestically suggestive, this phenomenon cannot be supported in our case because of the lack of immunoblotting and enzyme-linked immunosorbent assay (ELISA) to confirm changes in the antibody profile [10].

Pemphigus vulgaris represents a therapeutic challenge with multiple classes of drugs being available to control this condition, such as corticosteroid therapy, immunosuppressive therapy, targeted biologic therapy, intravenous immunoglobulins, or plasmapheresis procedures (Table 1) [11,12,13]. Corticosteroid therapy (topical and systemic) remains the first line of treatment for these patients regardless of the severity of the disease, with the other therapeutic classes being accessed in case of poor control or disease progression with corticotherapy. Moreover, due to administrative issues, such as limited access to these therapies in some countries (iv immunoglobulins or biologic therapy) or the lack of standardized protocols (in the case of plasmapheresis or iv immunoglobulins), explain once again the need for first-line administration of affordable therapies, whose administration is well standardized by protocols and studies (corticosteroids, dapsone, immunosuppressive therapy with azathioprine or mycophenolate mofetil) [11].

**Table 1:** Therapeutic lines used in the treatment of pemphigus vulgaris and their main characteristics

Treatment line	Type of treatment	Characteristics
1	Corticosteroids (local)	Only in mild forms.
	Corticosteroids (systemic)	High efficiency, but numerous adverse reactions on long-term therapy
	Dapsone (alone or with corticosteroids)	Efficient steroid-sparing agents; rare or no adverse reaction on long-term therapy
	Rituximab	Standardized protocols, prolonged remission in a high percentage of treated patients; high costs
2	Plasmapheresis	Requires combination with other types of therapy (this combination increases the risk of infection); costs are low, but there are no standardized protocols that specify the number of cycles and the rate of administration
3	Immunoglobulins iv	High costs and no standardized protocols that specify the number of cycles and the rate of administration; the exact mechanism by which they work are unknown

After approximately three months of treatment, the patient presented with exacerbation of his cutaneous-mucous lesions, an event that could be the result of the decrease in corticosteroid doses in the tapering process. In addition, viral diseases, including COVID-19, can be triggers for autoimmune reactions [14]. There are several ways to stimulate autoimmunity: molecular mimicry and exposure to autoantigens (bystander activation) with the subsequent expansion of autoreactive T cells through the phenomenon of epitope spreading. Molecular mimicry is when there here may be a similarity between viral and host antigens causing the identification of autoantigens and destruction by the immune system. Bystander activation involves the possibility when there is no antigenic similarity, but cytolysis causes an increased amount of antigens that exceeds the capacity of phagocytosis, autoantigen exposure, and generation of autoreactive cells. After either of these two processes, epitope spreading is the amplification of the immune response that leads to the targeting of autoantigens different from the first targeted antigen, and the development of autoantibodies [15].

With the discovery of the molecular mechanisms underlying the disease, new targeted therapies were introduced and a gradual change in the management of the patient with pemphigus vulgaris was observed. Rituximab, a chimeric type I monoclonal anti CD20 antibody, is an effective therapy in the management of the patient with pemphigus, by targeting B lymphocytes and decreasing the formation of anti-desmoglein autoantibodies, molecules with a central role in the pathogenesis of the disease. It was used as intravenous therapy initially for refractory forms of the disease, but recent studies are investigating the benefits of this therapy as a first-line treatment. They showed that rituximab may decrease the duration of the consolidation phase of therapy and help decrease corticosteroid doses [16]. Early initiation of rituximab therapy has been associated with better response rates, and according to long-term follow-up data 35% to 45% of patients remain in complete remission after stopping therapy, and relapse may be caused by persistent clones of autoreactive B cells [6]. Systemic therapy with rituximab involves the risk of side effects, the most commonly reported being iv therapy-related and infectious [17]. In this case, the patient developed an immediate iv therapy-related reaction with urticarial lesions and angioedema that disappeared at the cessation of therapy, followed by the iv administration of corticosteroids and antihistamines. At that time, the possibility of desensitization to rituximab in this patient was considered to benefit from an effective therapy and to decrease the doses of systemic corticosteroids, but this alternative was abandoned due to the patient's refusal.

The present case represents a therapeutic challenge, considering the therapeutic limitations given on the one hand by the precarious financial situation (which prevented access to iv immunoglobulin therapy) and on the other hand by the impossibility of relapse control using targeted therapy with rituximab (because of therapy-related side effects). 

Limitation of therapeutic options

In front of a young patient who needs therapy for a chronic pathology, considering the adverse effects of long-term corticosteroid therapy, limited efficacy, and important adverse reactions of standard immunosuppressants in the management of pemphigus vulgaris, a therapeutic option must be decided. Given these events, we decided to initiate therapy with dapsone, a steroid-sparing agent, with previous testing of serum glucose-6-phosphate dehydrogenase (G6PD) activity to avoid hemolytic adverse events. Dapsone, traditionally used in the treatment of leprosy, may be an adjuvant therapy in the management of pemphigus. Doses between 50 to 200 mg/day of dapsone also determine the possibility of decreasing or eliminating the doses of systemic corticosteroids with disease control and minimizing the risk of viral infections [18]. This therapeutic strategy led to a slow, but progressive recovery over the next several months. For five months after the discharge date from the hospital, the patient has received dapsone therapy and also a tapering dose of methylprednisolone (4 mg/day) without recurrence of the disease. After five months without signs of disease activity, it is decided to eliminate corticosteroids from the treatment regimen, keeping dapsone as the only treatment, without the appearance of new lesions at the last presentation. Moreover, in the context of the current pandemic, in which conventional immunosuppressive treatments and targeted therapies lead to altered immune mechanisms and an increased risk of COVID infection, dapsone therapy not only is not a risk factor but has been shown to even had a protective role against severe forms, possibly due to its antineutrophilic effect [19].

## Conclusions

Pemphigus vulgaris is a chronic autoimmune disease with significant morbidity and mortality in the absence of proper treatment, and potentially fatal before the introduction of systemic corticosteroids in the management of these cases. Although systemic corticosteroids are the mainstay of treatment, finding the most effective steroid-sparing agent represents a challenge for clinicians. The understanding of the etiopathogenesis of the disease has led to the increased use of targeted therapies as a first-line, which reduce the risk of side effects of systemic corticosteroids and cause an earlier clinical response. However, an individualized approach of each patient is required, considering patient comorbidities, even the risk of infection, financial status, and compliance with treatment. Treatment may represent a double-edged sword in this case where the patient developed COVID-19. In particular, the new targeted therapies (rituximab) cause a spectacular clinical improvement in these patients, but they increase the risk of SARS-CoV-2 infection which in turn can stimulate autoimmunity and exacerbate the underlying disease. Although not a first-line therapy and rarely used as a single therapy (often associated with corticosteroids or immunosuppressive therapy), dapsone may be an effective treatment option in those patients in whom first-line therapies have failed or cannot be accessed due to financial reasons. In conclusion, early diagnosis and tailored treatment are vital for the management of these cases, reducing morbidity and mortality.
